# Building Community around Food in Urban Spaces: Considering Connecting and (Re)connecting to Indigenous Foods in the City

**DOI:** 10.1016/j.cdnut.2026.109447

**Published:** 2026-07-22

**Authors:** Megan Mucioki, Tara L Maudrie, Julie A Beans, Ruby Fried, Kerry Hawk Lessard, Michelina Schach, Maurice Hall, Peter Attarian

**Affiliations:** 1 Department of Environmental Sciences, Emory University, Atlanta, GA, United States; 2 School of Social Work, University of Michigan, Ann Arbor, MI, United States; 3 Department of Health Behavior and Health Equity, School of Public Health, University of Michigan, Ann Arbor, MI, United States; 4 Department of Nutritional Sciences, School of Public Health, University of Michigan, Ann Arbor, MI, United States; 5 Southcentral Foundation, Anchorage, AK, United States; 6 Institute for Circumpolar Health Studies, University of Alaska Anchorage, Anchorage, AK, United States; 7 Native American LifeLines, Baltimore, MD, United States

**Keywords:** urban Indigenous food sovereignty, food sovereignty, traditional foods, community, American Indian and Alaska Native People, Anchorage, Baltimore

## Abstract

**Background:**

Today, well over half of American Indian and Alaska Native (AI/AN) People in the United States live in cities, a growing trend since the mid-20th century. Indigenous food sovereignty is a concept and process rooted in Indigenous self-determination of food, health, and nutrition.

**Objectives:**

Although often considered in the context of rural or reservation-based AI/AN communities, there is burgeoning consideration of Indigenous food sovereignty and its unique manifestation in cities. In this paper, we ask: How are AI/AN People making and building community around food in urban spaces? How does this contribute to our understanding of Indigenous food sovereignty in process and definition?

**Methods:**

Our study is informed by lived experience and/or planning discussions of authors from 2 urban spaces in the United States: Anchorage, Alaska, and Baltimore, Maryland. Authors include Indigenous and non-Indigenous researchers and community leaders engaged in community-based participatory research. Most authors have personal experience living in Anchorage or Baltimore.

**Results:**

We found unique or amplified challenges, such as high cost of living, poor access to land, smaller or different community and family networks, more challenging access to traditional foods, and centering AI/AN community ethics and values in urban policies. However, AI/AN innovation counters urban challenges to Indigenous food sovereignty, and community is a keystone component of these innovations.

**Conclusions:**

We conclude with recommendations for future research, outlining important and impactful work to be done to support urban Indigenous food sovereignty. This includes quantitative and mixed-methods studies, social networks analysis, and consideration for environmental health related to traditional foods and Indigenous land stewardship in cities.

## Introduction

Today, 8.8 million American Indian and Alaska Native (AI/AN) Peoples in the United States live in cities; AI/AN urban residents have increased 93% from 2010 to 2020, a 3-fold increase than rural AI/AN population growth [1]. Since before settler arrival in the United States, AI/AN Peoples have lived in and claimed cities, meaning that the history of all cities in the United States cannot be separated from the history of AI/AN Peoples [2]. Traditional foods and relational cultural components are central to AI/AN lifeways, health, and well-being. Traditional foods are defined as foods that have generational and cultural significance for AI/AN Peoples. These foods include wild-harvested or domestically reared plants, fish, and animals [3]. However, the term “food sovereignty” itself is more recent [4]. La Via Campesina [5] established a shared definition for food sovereignty in 1996 in protest of global, neoliberal food systems and stemming injustice to small farmers and food harvesters, inclusive of Indigenous Peoples. Over a decade later, food sovereignty was (re)defined and applied as a term to describe the efforts of Indigenous Peoples in North America to determine their own food systems and well-being and is often used in response to the ongoing colonization of their food, land, and health and well-being [3]. Maudrie et al. [4, p. 3] states: “Indigenous conceptions of food sovereignty expand on the values offered through the broader food sovereignty movement (e.g., agency, rights-based approach to food) by centering the value of food to Indigenous Peoples in holistic well-being (physical, social, spiritual, emotional, and intellectual), in being accountable relatives to our food systems, and emphasizing responsibility to engage with one another and our food systems with reciprocity and respect.”

As the abovementioned description emphasizes, Indigenous food sovereignty connects to agency and rights in the broader food sovereignty movement while centering the holism, relationality, responsibility, respect, and reciprocity that are central to the Indigenous connection with food. Although described in one way here, food sovereignty is based on self-determination with a proliferation of definitions and applications that depend on the actors [6]. Definitions and applications have not been clearly attributed to rural or urban places, although the majority of Indigenous food sovereignty studies are informed by work with rural communities [7]. However, this is changing; for example, Maudrie et al. [8,9] developed Indigenous nourishment scales based on discussions with AI/AN Peoples in Baltimore, Maryland; Minneapolis, Minnesota; and St. Paul, Minnesota. Of the research that has been done on urban Indigenous food sovereignty, there is geographical and regional clustering in Ontario, Canada (10 studies from this region and 6 additional from other locations in Canada) [[10], [11], [12], [13], [14], [15]]. There are only 5 articles focused on urban Indigenous food sovereignty in the United States [[16], [17], [18], [19]]. A review article on urban AI/AN food security found a similar geographic trend in research ∼10 years ago [7]. Although AI/AN Peoples across metropolitan areas are increasingly engaging in initiatives related to Indigenous food sovereignty on the ground [7], practice and policy informed by research in this area is fairly new. Furthermore, the data gap that exists in general on AI/AN Peoples at the population level is amplified in urban AI/AN populations [20]. However, in the United States, organizations like the Urban Indigenous Health Institute in Seattle, Washington, and the Southcentral Foundation (SCF) in Anchorage, Alaska, are working to center Indigenous data sovereignty while providing information and data for urban AI/AN populations that can aid food and health initiatives, among other things [19,21].

In this study, we add to the understanding of urban Indigenous food sovereignty, inclusive of traditional food security (access to, availability of, and utilization of traditional foods and essential cultural institutions [22]). We add to the existing literature by expanding the geographic scope, presenting experiences in 2 cities with very different urban ecologies yet overlapping findings in the importance of community, and identify urban Indigenous food sovereignty research needs that will be the impetus of future work. Our consideration stems from collective planning work and lived experiences in 2 urban spaces in the United States: Anchorage, Alaska, and Baltimore, Maryland. Authors include Indigenous and non-Indigenous researchers and community leaders engaged in community-based participatory work. Most authors have personal experience living in Anchorage or Baltimore. In this paper, we ask: How are AI/AN Peoples making and building community around food in urban spaces? How does this contribute to our understanding of urban Indigenous food sovereignty in process and definition?

### Communities

#### Anchorage, Alaska

Anchorage is in southcentral Alaska at the end of the Cook Inlet on the traditional homelands of the Dena’ina Athabascan People and the Native Village of Eklutna (Idlughet Qayeht’ana). There are 229 federally recognized Tribes in Alaska, and AI/AN Peoples make up >20% of the state’s population. In 2025, almost half of the state’s population (∼406,633 people) and ∼37% of the AI/AN population lived in the Anchorage and Mat-Su metro area [23]. Anchorage is an intertribal space, with people from many different Alaska Native Tribes that often engage with each other; Alaska Native People in Anchorage are 14% Athabascan, 24% Inupiaq, 14% Aleut, 11% Yup’ik, and 37% of other AI/AN descent [24]. Residents, regardless of racial or ethnic background, can catch many species of fish (including salmon) and pick berries (raspberries, blueberries, crow berries, and cranberries) and traditional greens within city limits or a few hours’ drive [23]. There is a large geographic discrepancy in annual wild food harvest, with 276 pounds (125 kilograms) per person per year in rural areas vs. 19 pounds (9 kilograms) per person per year in urban areas [25]. AI/AN Peoples in Anchorage, and in some cases throughout the state, are supported by Anchorage-based organizations such as SCF, Alaska Native Tribal Health Consortium, Alaska Native Health Board, Cook Inlet Tribal Council, Alaska Native Heritage Center, and Alaska Federation of Natives. Many of these organizations have health- and food-related services and programming.

Over the past 3 years, 3 of the authors have worked on a National Science Foundation planning effort to capture lived experience and community building needs related to urban traditional foods security and sovereignty in Anchorage [26]. This project informs part of this paper. MM is of Euro-American ancestry and currently resides in Atlanta, Georgia, the original homelands of the Muscogee (Creek) Nation. She has been involved in research in Alaska since 2020. RF is of Euro-American ancestry, was born and raised in Alaska, and continues to live in Anchorage, Alaska. She has been engaged in research with Alaska Native People for the past 10 years. JAB’s family is from the lower Yukon in Southwestern Alaska and the Oneida Nation of Wisconsin, and she was born and raised in Alaska. She has engaged in research in service of Alaska Native People for over 11 years.

#### Baltimore, Maryland

The Baltimore Native community is a dynamic and resilient community, consisting of ∼35,000 AI/AN Peoples living in Baltimore and the surrounding service area [27]. Many members of the Baltimore Native community hail from local Tribes such as the Piscataway and Susquehannock, whose ancestral homelands include Baltimore and the surrounding areas. Notably, Baltimore has the largest population of the federally recognized Lumbee Tribe outside of North Carolina. Other AI/AN Peoples lived in the Baltimore area for a period of time, eventually moving away but maintaining a connection to the community. In recent years, the Baltimore Native community has struggled for visibility within Baltimore and the surrounding areas, often fighting for disaggregation of data and recognition within their city [28]. In part, this invisibility may be due to the contemporary geographic spread of the Native community within Baltimore and the surrounding areas. This geographical spread of the Baltimore urban Native community has made it difficult for the community to maintain land-based projects like community gardens. Food is an especially powerful connector in Baltimore, offering a meaningful and accessible pathway back to culture for many AI/AN Peoples who have experienced disconnection or historical trauma. East Coast Tribes in particular have a complex and painful history with settler colonialism due to earlier and sustained occupation of their homelands by settlers. East Coast Tribes endured devastating losses from infectious diseases and violent conflicts, and in many cases, their traditional foods were weaponized as a way to control them and force assimilation. As a result, traditional food knowledge in some East Coast Tribal communities has become extremely limited. AI/AN People living in Baltimore, like many urban AI/AN populations, are disproportionately affected by opioid overdoses and forms of substance misuse [29] and the HIV/AIDS epidemic [30]. The community-based participatory research (CBPR) work and social advocacy from the community has revealed that disconnection from culture has impacted the ability to feel well or whole. In spite of these challenges, the Baltimore Native community is a vibrant community that frequently speaks out against social injustices, while gathering to share stories, foods, and practices from their respective homelands.

Author TLM, an enrolled citizen of the Sault Ste. Marie Nation of Chippewa Indians, lived in Baltimore, Maryland, for 3 years while in graduate school at the Johns Hopkins Bloomberg School of Public Health. During her time physically living in Baltimore, she developed a strong working relationship with the executive director and staff of Native American LifeLines (NAL), a title V Indian Health Services outreach and referral center. As a member of the community, she developed close relationships with many community members and frequently attended community events, including volunteering at COVID-19 vaccination clinics and attending initiatives like “Keeping the Heartbeat Strong,” an event that combined drum making and naloxone (Narcan) training. Author KHL is a descendant of the Fort Peck Assiniboine Tribe and has lived in Baltimore almost her entire life. Throughout her life, KHL has been personally and professionally involved in the Baltimore urban Native community through cultural and community health work. KHL is an applied medical anthropologist and did her undergraduate work at Florida Atlantic University and her Master of Applied Anthropology at University of Maryland College Park. Throughout her undergraduate and graduate work, she focused on adapting HIV prevention interventions for urban AI/AN populations. Currently, she is the Executive Director of NAL and is deeply involved with national advocacy for urban AI/AN communities.

## Methods

The work presented in this paper stems from projects focused on the lived experience of community members and authors and planning for future research with said communities. Work in Anchorage and Baltimore were done separately with differing methods but similar goals, approaches, values, and ethics. Authors came together on this topic after each completed their inquiries in an effort to provide a combined perspective on urban Indigenous food sovereignty that includes 2 very different cities in the United States, yet with findings and experiences that overlap in some respects. Each case shared was not constructed in the typical style of social science research inquiry but was instead meant to share lived experience and inform future research in these places and broadly on urban Indigenous food sovereignty in the United States. The Anchorage work was a planning project, and the work in Baltimore was pulled from multiple different studies as part of TLM’s dissertation research as well as the lived experiences of TLM and KHL as Indigenous Peoples living in Baltimore. Thus, the results do not exactly mirror each other in content and style, given different but parallel project trajectories and the style and experiences of the authors.

Over 3 years, MM, RF, and JAB led a planning effort to understand the challenges and assets related to Alaska Native traditional foods security in Anchorage, Alaska. This work was reviewed by university-based institutional review boards (IRBs) and the Alaska Area Indian Health Services IRB; both determined that planning discussions did not require IRB review and approval but were permitted to be shared in support of future directions. The authors led 2 discussion groups in fall 2023—at the SCF Traditional Foods Gathering with 30 people (28 September, 2023) and the Alaska Food Policy Council Festival and Conference (11 November, 2023) with 40 people—to understand issues related to traditional foods access and consumption in Anchorage. The team also engaged in informal conversations at the Intertribal Agriculture Council Alaska Regional Summit (8–9 November, 2023) and learned from speakers at the Native American Nutrition Conference (10–12 September, 2023) reaching ∼15 to 20 more people. Discussions were guided by the following 5 big picture themes: methods of accessing traditional foods in urban spaces; challenges to accessing traditional foods in urban spaces; programs that support traditional foods access and use; programs people wished existed to support traditional foods access but did not; and youth-related needs and challenges related to traditional foods in Anchorage. The findings were subsequently presented with more limited discussion at the Intertribal Agriculture Council Annual Meeting (10–12 December, 2024) and the second SCF Traditional Foods Gathering (14 August, 2025). Further discussion at these 2 meetings helped to enrich initial themes and add detail and further lived experience to our findings. Discussion groups collectively included ∼80 people; over half self-identified as Alaska Native or as non-Alaska Native People working for an Alaska Native-serving organization. Many participants had experienced moving to Anchorage at some point in their lives, which often shaped the discussion and their responses. Authors also participated in working group calls and small group or one-on-one discussions with people who conduct food-related work in Anchorage and serve Alaska Native populations. At each meeting, detailed notes were taken by authors and research support staff. Notes were read closely by MM, RF, and JAB, and themes were selected, with detailed examples from participants grouped into each theme.

The perspectives and lived experiences represented in the case study of the Baltimore Native community are a direct result of personal and professional observations, connections, and engagement with members of this community. These observations are also connected to a number of CBPR studies led by TLM [8,9,31,32]. During her master’s program, author TLM worked with NAL to conduct a mixed-methods CBPR study to understand the effects of food insecurity. This study included a quantitative survey of over 200 urban AI/AN adults and in-depth interviews with 11 participants from the quantitative study to better understand their experiences with food insecurity and food sovereignty [31]. Author TLM supports the Baltimore Native community through personal service and by regularly engaging community members in her research through events that combine seasonal gatherings, such as the spring equinox or summer solstice, with research dissemination and co-interpretation of results. The perspectives presented in this paper are derived from TLM’s work and personal connection and experience with the AI/AN community in Baltimore. KHL’s work is informed by her graduate work and her involvement with a Center for Substance Use Prevention grant, which braided together community-expressed needs, Indigenous knowledge, and Western research methods that were applied to improve the health status of urban AI/AN People in Baltimore. This work translated into her (KHL) role as Executive Director of NAL of Baltimore, a role that enables her not only to deliver services to the community but also allows her to advocate locally and nationally for urban AI/AN health needs that are experienced in communities like Baltimore.

## Results

### Anchorage, Alaska

#### Food as a connector

Although the 229 federally recognized Tribes in Alaska reflect distinct cultural differences, food is a cultural component that creates connections in Anchorage. Across discussion groups, participants expressed the desire to increase their knowledge of the land, share knowledge with others, and build community. People emphasized the need to maintain community sharing even in a new place (for those who may have moved from rural villages) and to continue to “give with your heart.” They emphasized the importance of maintaining connections with Elders, who serve as vital knowledge keepers to care for and learn from, particularly regarding traditional food sharing and practices. People mentioned that institutions like the Alaska Native Charter School and the Alaska Native Medical Center provided organized opportunities to engage with traditional foods and other people.

#### Changing family/kinship networks

Fragmented family structures were described by those who moved from their often rural or remote homelands to Anchorage. Moving to a new place can change family connections, with distance making it harder to learn food harvesting, processing, and land-based practices, as well as reducing access to cultural gifts and traditional food sharing. For example, “When I first moved to Anchorage, I did not know how to feed my family other than the store,” or “Took more than 5 years to figure out where to get fish and where to pick berries.” Some people in Anchorage said they lacked family to provide for them when they could not access traditional foods themselves but had a desire to consume these foods. Some felt lonely eating traditional foods by themselves, and some went so far as to say they would not eat certain foods alone because that is not how they are supposed to be consumed. Discussants also reported having to scale down food preparation; they had fewer people to cook for and share foods with than in rural spaces. Having to travel back and forth from urban to rural areas to access traditional foods is an economic burden and not sustainable for everyone. However, this is an important seasonal activity for many people and is an important way to access traditional foods at a volume and diversity that is not possible in Anchorage. Traveling to rural places also helps sustain ties to family and friends that do serve as support for urban communities. Often, people traded traditional foods with rural residents in exchange for nonfood and grocery items not easily accessed in rural Alaska.

#### Emerging networks and reciprocity

Despite these changes, some participants noted that moving to the city provided opportunities to reconnect with extended family members with whom they previously had limited contact. This was also suggested as a way to stay close to cultural food practices and stay or become embedded in social networks of people who share the same values and practices around traditional foods. People said things like “Get to know who your relatives are or who they can be.” “Know your aunties and uncles—stay connected with family and get connected with family you do not even know about.” This could mean making connections with people that are not biological kin but serve as a familial-like network or contacting family they had not previously met. Part of the process of engaging in these networks is giving and reciprocity. One person said: “Trust in giving.” Building community involves fostering reciprocal relationships that strengthen social networks and essential ties to traditional food access and community in the city. Connecting with others in Anchorage who already hold these knowledge and experience is essential and desirable, but this is a connection many people were still seeking or taking the time to foster. Some people used online platforms to share knowledge and foster connection.

#### Access strategies and barriers (including regulations)

Discussants described challenges faced in accessing and consuming traditional foods. People wanted to know more about wild food and land regulations in Anchorage. People were often scared to go out by themselves and desired other people to join for personal safety and camaraderie. Others were not sure which plants were edible in the region, and some preferred traditional foods, such as seal or caribou, that were not available in the Anchorage area but accessible in their home villages. People said it was challenging to teach themselves or ask for help. Some viewed it as impolite to ask for help and thought it customary to wait to be invited or engage in social networks.

Participants were also worried about potential toxicities in urban areas through contamination of soils and waters. The influx of dipnetters and fishing guides on the Kenai, Kasilof, and Copper rivers was noted as an issue affecting salmon access, specifically king salmon. There are strict and often debilitating regulations on foods like clams, where heavy harvesting has led to very low limits or complete closures in some years. The privatization of land has also limited access to traditional foods. There are often unethical and/or culturally discordant harvesting practices exhibited by non-Indigenous Peoples in the area; respondents shared that this contributes to not getting enough salmon from the Kenai River or enough berries. Harvest limits and overharvesting result in more time and money spent on multiple or longer trips to harvest salmon and berries. People have observed that many berry harvesters do not follow Alaska Native cultural norms, such as only harvesting one-third of the berries on a plant or not picking all the berries in an area.

Beyond individual efforts, participants noted a lack of community support for gathering and processing in urban areas. With a larger and more dispersed population, communal harvesting practices are less intuitive, lacking the shared ethic found in rural villages where most residents are raised with Alaska Native harvesting values. The taste of certain foods like salmonberries and salmon differ from place to place and, oftentimes, when people move to Anchorage, they find the tastes to be different and, maybe in some ways, less preferred than the tastes from their home village. Gathering traditional foods in urban areas also means adapting the methods of traditional processing, like drying or smoking fish, which necessitates new methods for processing that rely on less or different types of space (e.g., outdoor drying racks vs. smaller, indoor spaces). It is also expensive to gather traditional foods in the Anchorage area. Traveling to harvest areas requires long drives and/or boat access. This can be costly and often requires unpaid leave from work because subsistence leave is less common in urban Alaska than in rural locations.

Some participants reported fear of hosting potlucks in public spaces in Anchorage. One person described public officials shutting down a gathering because of regulations around sharing non-commercialized foods and legal definitions of “safe” food. Others recounted having the fire department called for smokehouse use. Participants felt that municipal codes were outdated and needed updating, calling for clearer channels to report discrimination and noncompliance. There is a broader need for public institutions to better understand traditional food practices and their cultural significance to Alaska Native People.

Anchorage demonstrates how Indigenous food sovereignty and traditional food security are not lost in urban transitions but are reshaped—through new community ties, reciprocity, and adaptive food practices. Alaska Native People are actively working to curate and strengthen these elements under conditions of significant structural and regulatory constraint.

### Baltimore, Maryland

#### Access strategies and barriers

We often hear from those who have relocated to Baltimore that they are unsure how to teach their children about their specific traditional foods and food practices when they are so far away from their homelands. Although Elders within the community are excited to share their food teachings and practices with youth, without adequate land to demonstrate and share these practices, passing on this knowledge has been difficult in the past. However, these important indicators of AI/AN food system well-being are rarely captured in food systems research and evaluation efforts in Baltimore. Furthermore, access to land and the Baltimore weather make it challenging to implement traditional and ancestral food practices, given that until recently, the main Native health organization in Baltimore, NAL, did not own their own building. The humid hot summers in Baltimore are not typically conducive to growing foods evolved to grow in drier or cooler climates, including the traditional foods of many southern or northern AI/AN Peoples. Despite the growing popularity of foraging within dominant society, community members have expressed that stopping to pick something on the side of a road or in a public space might be viewed as odd or unacceptable behavior [32]. Within Baltimore, public spaces like parks are often treated with pesticides or are used for pet walking, making it unsafe and undesirable to gather foods or medicines.

Approximately 14% of AI/AN families and 20% of AI/AN individuals in the Baltimore service area live at or below the poverty level, compared with non-Hispanic White families and individuals, who experience poverty rates of 3.6% and 6.6%, respectively [27]. The strained socioeconomic status of many AI/ANs within the Baltimore community limits their ability to access affordable housing, safe spaces to exercise and spend time outside, and affordable and healthy foods. Throughout our years of community engagement, we have heard from community members that many of them live in what has been termed food apartheid, meaning that structural inequities and systemic inequalities created and perpetuated by racism and segregation limit their ability to access safe and healthy foods [33]. Many of our community members purchase their foods from corner/convenience stores, gas stations, and cheap carry out food restaurants because that is what is most readily available in their neighborhoods.

#### Strong desire for (re)connection with traditional foods and culture

Yet, in spite of these challenges, there is a strong and growing hunger for reconnection with traditional foods and food practices. The intertribal nature of the Baltimore Native community offers a vibrant and welcoming space for sharing foods, food knowledge, and cultural practices. Community gatherings frequently center food as a way to bridge differences, build relationships, and celebrate cultural diversity across Tribal affiliations [34]. Community members often express joy in sharing and learning about one another’s traditional foods; for example, comparing salmon and whitefish preparation methods can spark dialogue, connection, and knowledge generosity across Tribal nations. Elders relish sharing their food stories from their homelands with youth, who listen attentively, asking unique questions and sharing observations to connect their experiences with their Elders.

Recently, NAL acquired their own building and land and is working to plan spaces for traditional healing to grow medicines and foods. In the past, community members have expressed a desire to be able to grow culturally significant foods and medicines from their homelands (e.g., Navajo/Dine People and blue corn). Until 2025, NAL has rented office space and had limited access to land to realize these community requests. Even today, with their new building, which does have the ground-level outdoor space, there are strong concerns about soil quality on the ground level because of the presence of a cleaning chemical factory nearby. To ameliorate these concerns and threats from pests like cockroaches and rats, NAL is planning to grow these medicines on a rooftop garden. In December 2023, staff and leadership from NAL visited the American Indian Community Housing Organization to learn from their efforts to house and care for urban AI/AN relatives in their community. Through this visit, they learned about how rooftop gardening has impacted the urban AI/AN community in Duluth and plan to replicate and adapt their work to fit the context and needs of the Baltimore AI/AN community. NAL is also working with the chief health officer of the Native American Health Center in Oakland, California, to better understand how to overcome the challenges of growing medicines and foods in an urban environment. Working to grow medicines in a deeply urban environment requires thoughtful and intentional planning to ensure that medicines and foods are safe and protected from poor soil quality and pests. As many AI/ANs within the Baltimore community yearn for their homelands, NAL is working to provide a bridge to ensure AI/ANs have access to culturally significant foods through community gatherings.

## Discussion

In this discussion, we highlight elements and nuances of urban food sovereignty that we find important when developing an understanding of the process of and priorities in supporting urban Indigenous food sovereignty. We recognize this as a starting point, with the intention of developing further research and outreach on this topic; we conclude this discussion with recommendations for future directions for our own work and that of others. Traditional foods are central to food sovereignty in both rural and urban spaces, although urban environments present distinct challenges. Limited land access, difficulties building and sustaining community, smaller family and social networks, reduced access to traditional foods, policies that do not center AI/AN values, and persistent socioeconomic disparities all create significant stressors. In response, these conditions spur adaptation and innovation and, in many cases, strengthen community cohesion as a means of countering urban threats to Indigenous food sovereignty and cultural continuity.

### Amplified Western values that challenge community and AI/AN values

Some urban settings amplify Western values and practices of isolation/individualism, capitalism, commodification, and linear progression in ways that can aggravate health, food security, and wellness [35]. In Anchorage, for example, there was discussion that eating traditional foods home alone was not a typical practice and for some would not be done in isolation even if the foods were available. In Baltimore, gentrification challenged physical aggregation and connection among AI/AN households. Some research shows high levels of historical loss in urban AI/AN communities even when compared with reservation/rural communities [29]. Wiechelt et al. [29] found that urban AI/AN People in Baltimore had higher rates of thoughts related to historical loss and colonial trauma (e.g., loss of land or language, decreased trust in white people, and loss of family ties or generational connections) and historical loss-associated symptoms (e.g., depression, anxiety, and feelings of isolation) than AI/AN People living on a reservation in a rural environment. They hypothesize that: “factors related to enculturation such as cultural identity and cultural connection influence the trauma response and that reservation-based communities are more likely to have these connections” [29, p. 328]. This emphasizes the importance of cultural and community connections in Indigenous well-being, which may need greater support in urban spaces.

Particularly in Baltimore, health and food disparities were discussed. This is also a challenge in Anchorage, although study respondents did not mention it or prompts did not lend themselves to this discussion, and existing studies were not parsed by race [36]. Several other quantitative studies have found that food insecurity overall is higher among urban Indigenous households than rural Indigenous households [37,38]. Complementary qualitative studies have found factors that may explain these trends, such as notable economic struggles and higher cost of living [13,39], poor access to fresh and healthy food options, including traditional foods [7,40], fewer social safety nets [35], less access to shared harvesting resources [41,42], and less access to Tribal services [20]. Many of these challenges are also encountered by rural Indigenous populations but may be offset by stronger social networks and embeddedness in community, family, and culture [43]. Social networks and community are very important in urban spaces, as we emphasize below, but social safety nets may not be as strong because AI/AN individuals in cities are dispersed throughout a wider area.

The breadth and depth of people and cultural identities and practices in a city make it challenging to implement food harvesting practices that inherently support environmental sustainability and conservation. These include guiding values such as not taking too much, treading lightly, and respecting the land [44]. Ethics of harvesting traditional foods held by Indigenous Peoples are not always mirrored by non-Native foragers. Differences in how people engage with wild foods can affect harvest quantities, species and location selection, sustainability of practice, and food availability for groups like Elders or those with limited mobility. Urban foraging in practice and visibility may make it more socially acceptable broadly but can also put more pressure on resources and shift the context, values, and culture of a practice with Indigenous origins and connections to traditional foods.

### Cities present new ways and strategies for connecting with traditional foods

Indigenous innovation and adaptation can be a source of pride and self-esteem for urban Indigenous Peoples [45]. There is an inherent push and pull between challenges and resulting adaptations and innovations developed and enacted by urban AI/AN Peoples to maintain food sovereignty and traditional food security. Our study and others [46,47] have demonstrated that Indigenous Peoples in cities are consuming traditional foods, supported by Indigenous-led innovation and adaptation, and gaining access to more traditional foods is a desired goal. Specifically, 89% of Alaska Native mothers living in Anchorage reported preferring to eat more traditional foods than they can currently get, and 26% and 54% reported that their children aged <7 years had either eaten traditional foods within the past 24 h or past week, respectively [46]. Redwood et al. [48] examined change in traditional food use in Anchorage between 2004–2005 and 2015–2017 and demonstrated an increase in berry picking and cutting/smoking fish and meat over that time period. Traditional foods-related skills and knowledge do travel from place to place and sometimes from Tribe to Tribe. Even if traditional foods are not present, related skills and knowledge can still be applied and practiced on nontraditional foods to maintain proficiency until access to traditional foods is restored. Cities can be intertribal gathering spaces and places of cultural exchange, as in the case of AI/AN People in Baltimore learning about rooftop gardening from Indigenous People in Duluth and Oakland, as well as hubs for communities living both on and outside their traditional homelands. Food is a medium of knowledge and cultural exchange and urban Indigenous community building.

### Community is a keystone component of urban Indigenous food sovereignty

Community and social connections that support access to traditional foods through communal harvesting, being on land together, sharing food, and sharing food-related skills and knowledge are central to urban Indigenous food sovereignty. As mentioned, community in urban spaces can be expansive, including intertribal networks and strong personal connections with individuals who are not necessarily genetic relatives within and across cities. Likened to the foundation of self-determination in food sovereignty, the urban Indigenous community is very much determined by Indigenous Peoples themselves based on personal identity and relations, acceptance of community, and maintenance of and sustained connection to culture and kin inclusive of food that are essential to holistic health and well-being. Self-determination or self-identity aligns with how other studies [7,49] “define” urban Indigenous communities in their work. Our findings do suggest that community and social networks in urban spaces require more intention and support to develop and maintain; if they are absent or weak, the process of urban Indigenous food sovereignty has amplified challenges. They may also encompass a broader definition of community than rural places [45]. Having formalized structure and organization that support urban Indigenous community building seems to be essential. We see this potentially in the form of programs and workshops that offer (re)connection with traditional foods and skills [10,11,16,19], Urban Indigenous Food Banks [50], spaces for food cultivation of things like taro [17], seed saving and sharing [51], or leadership in land rematriation [18]. Indigenous-serving organizations in our focal communities, such as the SCF, NAL, or Baltimore American Indian Center, are central places for community building and connection with culture and food. The Baltimore American Indian Center offers semiregular culture classes, community connection opportunities, and annual events for both Native and broader Baltimore communities. NAL provides regular cultural and health programming, annual community events, and youth programming for AI/AN students K-12. The SCF leads a biannual Traditional Foods Gathering in Anchorage featuring a variety of workshops and speakers focused on knowledge transfer and skill building about traditional foods and medicine harvest and processing [19]. The Alaska Native Medical Center in Anchorage serves traditional foods, such as seal soup and caribou stew, to their patients and provides traditional food cooking and preparation training to Alaska Native chefs [52]. Although urban Indigenous-serving organizations often carry the task of providing culturally appropriate services and support for Indigenous Peoples, they can lack stable funding support and decision-making power, which makes sustainability and systematic change challenging [35].

Reciprocity is also a central ethic and practice to Indigenous food sovereignty and well-being in cities but again takes more intention to foster. Many studies have shown that food sharing is one of the primary ways that urban Indigenous households continue to access traditional foods. For example, among Alaska Native mothers in Anchorage, 87% reported sharing food with immediate family, 36% with extended family, 22% with friends, and 14% with other groups, such as church communities [46]. Moreover, those who reported >1 source of traditional food sharing reported eating significantly more traditional foods over the past year [46]. However, sharing most likely remains higher in rural areas, as evidenced by Smith et al. [53]: urban Inupiaq Elders reported 3 instances of food sharing compared with 55 sharing events reported by their rural counterparts over the same survey period. In Baltimore, AI/AN households experiencing food insecurity often receive food from family members [32]. Furthermore, community events in Baltimore offered the opportunity for food-related knowledge and skill sharing [32]. We suspect there are differences in sharing practices and abilities depending on the length of residence in urban spaces, although research has not been explicit about these trends yet. For example, generational urban residents may have more social connections within cities but fewer connections outside of urban spaces, which could shape the types of traditional foods they have access to. Although newer urban residents or residents that move between urban and rural spaces are more vulnerable to weaker community ties and gaps in place-based knowledge in cities, they may have more opportunities for traditional food access in rural spaces.

### Urban Indigenous food sovereignty: future directions and recommendations

The existing and budding research on urban Indigenous food sovereignty has been pivotal in beginning to spotlight Indigenous Peoples’ lived experiences, practices, needs, and preferences related to traditional foods in urban spaces. As we have shared, there is a growing body of literature that has presented parallel examples in cities such as San Francisco/Oakland, Minneapolis, or Portland, Oregon [16]. However, the literature on this topic in the United States is quite sparse, and in North America, it is highly clustered around Southern Ontario, motivated by the work of a few authors. There is much more that remains unknown about this topic, and research can play an important role in generating information and data to support Indigenous communities in cities and urban Indigenous food sovereignty (Table 1). To date, almost all of the published research has been qualitative in nature (an exception is [13]). This has been very useful in capturing lived experiences; however, we are missing information about broad trends at the population level for urban Indigenous Peoples. There is a need for quantitative research and mixed-methods research on these topics. There is also a need to broaden the places in which this work is being done, capturing more cities in the United States, while learning from and building upon much of the important work on this topic. Research may be helpful for understanding the barriers and challenges that come with community building in urban Indigenous spaces. Researchers may consider using social network analysis and food sharing analysis (this could also span rural areas) to understand existing and missing connections between community members and knowledge sharing (for a rural example, see [43]). We also recommend considering how differences in urban residency and generational connections to urban spaces impact household traditional food security as well as community related to food, knowledge, and skill exchange. Urban Indigenous food sovereignty studies should not only focus on urban spaces but also on urban to rural spaces when appropriate. Neufeld and Richmond [12] and Richmond et al. [13] have started to do this.

**TABLE 1:** Key recommendations of future directions in urban Indigenous food sovereignty research

• Expanded geographies and place-based work in cities in the United States
• Quantitative and mixed methods studies/data
• Collaborative work with urban American Indian and Alaska Native communities and organizations serving them
• Research that includes social network analysis and food sharing analysis to understand existing and missing connections between community members and knowledge sharing
• Research considering how differences in urban residency and generational connections to urban spaces impact household traditional food security as well as community related to food, knowledge, and skill exchange
• Inclusion of both urban and rural spaces, when appropriate, and considerations of the urban–rural continuum
• Environmental health considerations of soils and waterways and pathways of contamination accumulation in plants, animals, and fish to holistically understand the needs of the physical environment within the context of growing and harvesting foods
• Indigenous-led urban land restoration and stewardship

Cities often have heightened environmental health concerns with contaminated soils and waterways and denser areas of industrial work and traffic [54], concerns highlighted in our case studies. It might be useful for researchers to consider the quality of soils and waterways and pathways of contamination accumulation in plants, animals, and fish to holistically understand the needs of the physical environment within the context of growing and harvesting foods. Testing can be done for contaminants in the environment and harvested parts of plants to help shape practice around urban harvest and environmental health ethics. This should be done with great care to not create fear or stigma related to these foods but transparent information and practice related to safe harvest practice by working together with harvesters. This research should also consider what urban land stewardship and restoration looks like led by Indigenous Peoples in urban spaces and how land-based practices can be integrated into urban land planning and priorities.

Finally, researchers should partner and work with AI/AN communities and AI/AN-led organizations, as they are often the backbone or infrastructure of Indigenous communities in cities. Research should be informed by the experience of these organizations and aim to support existing and new program development.

## Conclusion

Urban Indigenous food sovereignty is an essential component of self-determination by AI/AN Peoples and communities in relation to food, culture, identity, and health and well-being in both rural and urban spaces. Although there is emerging research on this topic, much is still to be done, including a broader geographic understanding in the United States and more diverse and inclusive methodologies. In this paper, we identified and discussed community as one element of urban Indigenous food sovereignty, finding that it is a key component to traditional foods access and all that is connected to Indigenous food sovereignty in general but particularly in the context of cities. In the urban context, community can take on a broader meaning, and more attention and work may have to be needed to form social connections and engage in community around food. There is much that we do not know in terms of how AI/AN social and community networks and food sharing networks function in urban spaces and between urban and rural contexts. Expanding inquiry in collaboration with AI/AN communities and serving organizations will be important in learning more about Indigenous urban food sovereignty and supporting access to traditional foods in spaces where access can be very challenging but just as essential for restoring and maintaining health and well-being.

## Author contributions

The authors’ responsibilities were as follows – MM, TLM, JAB, RF: designed the work and led planning discussions and considerations of lived experiences; MS, MH, PA: supported literature review and paper content; MM, TLM, JAB, RF, KHL: wrote the paper; MM: had primary responsibility for final content; and all authors: read and approved the final manuscript.

## Data availability

Data summaries are available through cited works. Raw data are not available for sharing.

## Declaration of Generative AI and AI-assisted technologies in the writing process

The authors declare that no generative AI or AI-assisted technologies were used in the writing of this manuscript.

## Funding

The research in Anchorage was funded by a planning award from the National Science Foundation Arctic Social Sciences Program (NSF award #2306041).

## Conflict of interest

The authors report no conflicts of interest.
